# Management of Worsening Aortic Dilation and Insufficiency in a 20-Week Pregnant Woman: A Case Report

**DOI:** 10.1155/2011/483178

**Published:** 2011-07-10

**Authors:** Amy Shah, Johanna Schwarzenberger, Dorina Gui, Richard Hong, Angela Chen

**Affiliations:** ^1^Department of Obstetrics & Gynecology, University of California, Los Angeles, CA 90095, USA; ^2^Department of Anesthesiology, University of California, Los Angeles, CA 90095-7403, USA; ^3^Department of Pathology and Laboratory Medicine, University of California, Los Angeles, CA 90095, USA

## Abstract

Preexisting aortic disease can worsen during pregnancy as physiologic hemodynamic changes evolve. At a large academic institution, a patient with a remote history of vasculitis presented with a second trimester pregnancy with increasing aortic dilatation and aortic insufficiency. Extensive obstetric discussions encompassed maternal cardiac risks from continuing the pregnancy and fetal risks from maternal cardiac intervention. This patient desired termination of pregnancy to avoid further complications and to expedite surgical aortic repair.

## 1. Introduction

Strict prenatal care is necessary in pregnant women with underlying cardiac disease. Lewis and Drife reported cardiac disease to be the second most common cause of maternal death in the UK from 2000 to 2002 [1]. Recent evidence suggests an inherent risk of aortic dissection from pregnancy even without prior cardiac history [2]. Women with known worsening aortic dilation or aortic insufficiency have been shown to be at increased risk for heart failure, rupture, or aortic dissection during pregnancy [1].

Physiologic hemodynamic changes during pregnancy can complicate cardiovascular disease. Normally, as gestational age increases, cardiac output and intravascular volume continue to rise. In patients with preexisting aortic disease, these changes increase the risk for aneurysm formation or dissection. During labor, cardiac output elevates even further, potentiating the possibility of aortic disruption [3]. As a result, some of these patients undergo either termination of pregnancy or early cesarean delivery (depending on the patient's circumstances or preferences) to prevent further risk.

## 2. Case

A 32-year-old multipara presented to a large academic institution with a 20-week intrauterine pregnancy. She was diagnosed with polyarteritis nodosa as a child and was treated with both steroidal and nonsteroidal anti-inflammatory agents. After her teenage years, she had no relapses of her disease. However, in her previous pregnancy 3 years prior to this index presentation, the patient developed chest pain in the late mid-trimester. Evaluation at that time revealed aortic valve insufficiency and aortic dilation. She was intentionally delivered by preterm cesarean delivery at 30 weeks to avoid worsening hemodynamics should the pregnancy continue. The patient was placed on postpartum oral contraceptives but was again found to be pregnant.

**Figure 1:**
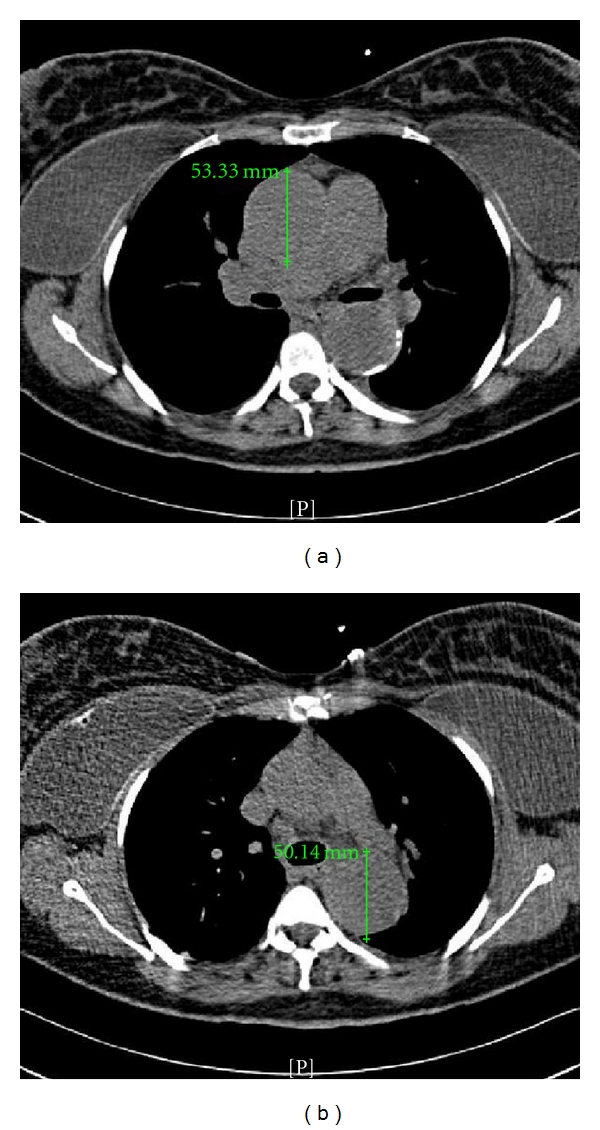
Radiographic changes with aortic dilatation. (a) Shows the ascending aorta measuring 5.3 cm in diameter. (b) Shows the descending aorta measuring 5.0 cm in diameter.

While receiving care by maternal fetal medicine specialists at an outside institution for this pregnancy, an MRI revealed worsening aortic dilatation that progressed from a 4 cm baseline up to 5.1 cm. The dilatation involved not only the aortic root, but also the level of the right pulmonary outflow tract, and a 15 cm segment of the descending aorta (Figure 1). Transthoracic echocardiogram additionally revealed new moderate aortic insufficiency with normal ventricular function, normal atrial sizes, and a normal mitral valve. The patient developed progressively worsening chest pain and dyspnea to the point that she had symptoms with her activities of daily living. After also being diagnosed with hypertension she was started on a beta blocker. Due to her declining cardiac status and the concern for acute aortic dissection or rupture, she was referred to our medical center for further evaluation at 20-week gestation.

The patient and her husband were extensively counseled about the risks of continuing pregnancy versus termination of pregnancy (TOP) via induction of labor or dilation and evacuation (D&E), with subsequent aortic repair. Anesthesiology, cardiology, and cardiothoracic surgery services were involved in the discussion with the patient and her obstetrician. A general consensus was made to proceed with a D&E abortion in an operating room where cardiothoracic surgery would be available should the patient experience intraoperative cardiac decompensation. 

On hospital day 1, the patient received an intrafetal digoxin injection and had cervical osmotic dilators placed. On hospital day 2, she received empiric antibiotics for spontaneous bacterial endocarditis prophylaxis and was taken to the operating room. General endotracheal anesthesia was administered in a similar fashion to a nonpregnant patient with an aortic aneurysm. Beta blockers, intravenous fluids, and a narcotic-based slow induction of anesthesia were performed to maintain hemodynamic stability and prevent sudden hypotension or reflex tachycardia [4]. Invasive lines were placed before the patient was asleep and blood products were in the room prior to the start of the case. With the cardiothoracic team on standby in the event of decompensation, routine D&E was performed under ultrasound guidance.

Postoperatively, the patient was admitted for cardiac monitoring. She developed an isolated, transient episode of chest pain within hours after her operation, which resolved spontaneously without EKG changes. A calcium channel blocker was added to her regimen to optimize her blood pressure. The patient was discharged home on hospital day 4. Prior to discharge, she was again counseled on using a more effective and long-term contraceptive method, including an intrauterine device, to prevent future pregnancy.

**Figure 2:**
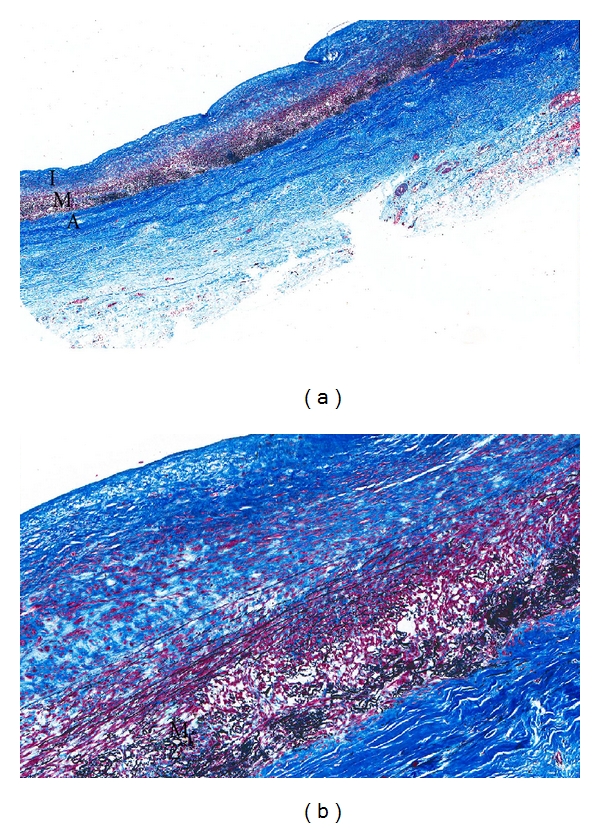
Microscopic views of the aorta after aortic graft repair. Magnification: 20x (a) and 100x (b). Aorta with marked medial degeneration with loss of elastic tissue and smooth muscle cells and with marked intimal and adventitial fibrosis (Trichrome-EVG stain). Magnification: 20x; detail 100x. (I: intimal layer; M: media; A: adventitia).

Within the subsequent months after the D&E, the patient underwent two separate operations to replace her ascending and descending aorta as well as insert a mechanical aortic valve. Her postoperative course was complicated by development of chylothorax, splenic laceration, and clostridium difficile colitis. The pathology report of her aorta revealed burnt-out aortitis supporting a diagnosis of prior vasculitis (Figure 2).

## 3. Comment

This case addresses the progression of aortic disease in a pregnant woman with a history of underlying vasculitis. Most cases of aortic dilatation in pregnancy involve Marfan syndrome (MFS). One study evaluated maternal and fetal outcomes of 14 pregnant women with MFS, who were followed with serial echocardiograms. Two women required surgical correction of the aortic aneurysm and replacement of the aortic valve; one within hours of vaginal delivery and another 8 weeks postpartum. The report observed that women with aortic dilation <40 mm tolerated pregnancy well with good maternal and neonatal outcomes, while women with cardiac decompensation or aortic dilatation >40 mm (as in the case of this patient) should be advised to avoid pregnancy [5].

While termination of pregnancy by D&E itself has inherent anesthesia and surgical risks, they have been shown to be much lower than the risks of full term pregnancy. For example, in the United States, the estimated maternal mortality after live birth is 7.06/100,000 compared to 0.567/100,000 after all pregnancy terminations [6], with the latter increasing to 8.9/100,000 in TOP > 21 weeks [7]. In contrast, the general average mortality rate after elective thoracic aortic repair on any average patient is about 3% [8]. For this patient, the risks of TOP were largely outweighed by the risks of continuing pregnancy given her symptomatic and worsening aortic dilatation. To further decrease the risk in this case, the cervix was prepared by osmotic dilator insertion the day prior to the procedure to decrease morbidity (particularly, cervical lacerations, uterine perforation, and incomplete abortion). Intrafetal digoxin injection promoted cortical bone softening and more pliable demised fetal tissue to facilitate complete evacuation [9]. Additionally, ultrasound guidance during the procedure maximized D&E safety [9].

Though prostaglandins can be used as alternative means of TOP, there are risks associated with the stress of labor in the setting of preexisting heart disease. During a normal pregnancy, cardiac output increases up to 51 percent during contractions [3] and an additional 10–20 percent immediately postpartum [10]. These substantial changes lead to increased aortic pressures in a patient with already worsening dilatation and insufficiency, increasing the risk for further damage. As a result, D&E remains a much safer option in this patient population.

Studies have shown that operative repair should occur prior to conception in patient with known aortic disease in order to prevent undue risk to mother and fetus. The use of cardiopulmonary bypass during pregnancy, with its resultant nonpulsatile maternal blood flow, exposes fetuses to uteroplacental insufficiency, causes deep hypothermia, and increases the risks for preterm labor and delivery [11]. Isolated cases have demonstrated the possibility of successful aortic valve and aortic arch repair early on in pregnancy. For instance, one study detailed a pregnant Marfanoid woman who underwent successful aortic valve and arch repair at 17 weeks (because of a 6.9 cm aortic dilation) and was able to deliver a healthy full-term infant [12]. Other reports note that, if cardiac surgery is indicated during pregnancy, and maternal condition permits, the optimal time is after fetal viability so a cesarean delivery can be performed concurrently [11].

With worsening aortic dilatation in our patient so early on in her pregnancy, there were concerns that the maternal condition may not tolerate expectant management until the fetus became more mature. There was some discussion of the best order for surgical interventions. Because the fetus was previable, we considered prophylactic aortic stent placement or aortic graft repair. Alternatively, immediate pregnancy termination would potentially mitigate the maternal risks for progressing gestation. Lacking established guidelines in the current literature, we followed the patient preference and proceeded first with the D&E. 

The main factor in decision making was the rapidly progressing aortic disease and potentially imminent cardiovascular event. Had the patient decided she wanted to continue the pregnancy, an extensive multidisciplinary approach would have needed to address the plan of care: temporizing measures such as an aortic stent placement coupled with risks of fluoroscopy and radiation to the fetus versus surgical correction involving cardiopulmonary bypass versus expectant management as long as feasible. These decisions by no means are static, and maternal and fetal health ultimately dictates plan of care.

Pregnancy causes physiologic hemodynamic changes that can be dangerous and can even be contraindicated in the setting of preexisting aortic disease. These patients should be counseled on effective and long-active fertility control options to prevent unwanted pregnancy. For the safety of these patients and future fetuses, maternal condition should be optimized prior to conception. In the event of pregnancy, arguments can be made that TOP is warranted to preserve maternal health and expedite repair, given that pregnancy will naturally progress the underlying disease and may potentially lead to maternal demise. While patients can undergo surgery during pregnancy, they do so with significant associated risks to the fetus. Ultimately, a multidisciplinary approach is necessary to determine each patient's wishes and the appropriate management for each case.
